# Mobile-Assisted intercultural competence development: The role of HelloTalk in Chinese EFL education

**DOI:** 10.1371/journal.pone.0328660

**Published:** 2025-07-17

**Authors:** Pei Yang, Ziye Yang

**Affiliations:** School of Foreign Languages for International Business, Hebei Finance University, Baoding, Hebei, China; McGill University, CANADA

## Abstract

This study investigates the impact of HelloTalk, a mobile-assisted language learning (MALL) platform, on the development of intercultural competence (IC) in Chinese English as a Foreign Language (EFL) students. A mixed-methods approach was employed, involving both quantitative surveys and qualitative interviews. Results indicate that the HelloTalk-based intervention significantly improved students’ IC, with the most notable gains observed in their knowledge of other cultures and intercultural communication skills. These findings suggest that mobile-assisted intercultural exchange offers a more effective approach to developing IC compared to traditional classroom learning. Qualitative data further highlight students’ positive perceptions of HelloTalk’s accessibility, flexibility, and the reduced communication anxiety it fosters. However, challenges such as difficulties in making connections and the potential for deception on mobile platforms were also identified. The study underscores the value of incorporating mobile technologies in EFL curricula to facilitate meaningful intercultural exchanges and improve students’ intercultural competence.

## 1 Introduction

In an increasingly interconnected world, intercultural competence (IC) has become a critical component of foreign language education, enabling learners to communicate effectively and appropriately across cultural boundaries [1–3]. For English as a Foreign Language (EFL) learners, IC is not only a supplement to linguistic proficiency but also a prerequisite for successful global communication [4,5]. In China, national educational frameworks such as *The National Standards of Teaching Quality for Undergraduate English Majors* have emphasized IC as a key learning outcome in English programs [6]. Despite this, many Chinese EFL classrooms remain limited in providing authentic intercultural experiences, with traditional instruction often failing to translate policy objectives into practical outcomes [7].

Emerging technologies such as AI, VR, and AR are indeed transforming language education and intercultural engagement worldwide. However, their adoption remains uneven, particularly in developing regions or underfunded institutions [8]. In China, a significant number of EFL learners – especially those in third-tier cities – still lack access to high-end technological infrastructure [9]. For these learners, mobile-assisted language learning (MALL) offers a more realistic and accessible solution. Mobile technologies provide affordable, flexible, and low-barrier platforms that allow learners to engage in language practice and intercultural interaction anytime and anywhere [10].

HelloTalk, a mobile application designed for real-time language and cultural exchange, provides Chinese learners with access to global users via text, voice, and video communication [11]. This is especially valuable given the restricted access to major global social media platforms like Twitter, Facebook, and WhatsApp in China [12]. Through contact with linguistically and culturally diverse speakers, HelloTalk offers learners authentic intercultural experiences that are often absent from conventional EFL classrooms. While existing research has explored its linguistic benefits [13, 14], little empirical work has investigated HelloTalk’s role in fostering intercultural competence, particularly in the Chinese context.

To address this gap, the present study examines the impact of HelloTalk on the development of IC among Chinese EFL students. Specifically, it explores the following two research questions:

(1) Does the use of HelloTalk significantly promote the intercultural competence of Chinese EFL students?
(2) How do students perceive HelloTalk for intercultural competence development?

By focusing on a widely accessible digital platform, this study aims to provide new insights into how mobile technologies can support intercultural learning in real-world educational contexts. The findings seek to inform future pedagogical practices and contribute to the broader discourse on technology-enhanced language education, particularly in contexts where access to advanced technology is limited.

## 2 Literature review

### 2.1 Concepts of intercultural competence

Intercultural competence has evolved from being synonymous with linguistic proficiency [15] to a complex construct involving attitudes, knowledge, and behaviors essential for effective communication across cultures [16,17]. Among widely cited frameworks, Byram’s model [16] identifies five core components – attitudes, knowledge, skills of interpreting and relating, skills of discovery and interaction, and critical cultural awareness – which have been foundational in IC scholarship [18–20].

However, Byram’s model [16], primarily developed in Western contexts, may inadequately capture intercultural dynamics in non-Western societies like China, where communication is more context-dependent and indirect. Scholars such as Chen and Starosta [21] have thus emphasized intercultural sensitivity and mindfulness in high-context cultures, arguing for more culturally adaptive frameworks.

This study adopts an expanded definition of IC as the ability to communicate appropriately and effectively across cultural boundaries by integrating knowledge, skills, attitudes, and reflective awareness. Building on existing models, we propose that mobile platforms like HelloTalk may serve as informal yet powerful ecosystems for dynamic IC development, particularly for learners with limited access to formal intercultural education.

### 2.2 Leveraging mobile technology in developing intercultural competence

Mobile-Assisted Language Learning (MALL) has become a prominent research field since the early 2010s, supporting flexible and interactive learning [22]. Initially focused on vocabulary acquisition and grammar practice [23,24], recent studies have explored MALL’s potential to facilitate intercultural communication. Platforms like Skype [25], WhatsApp [26], and Facebook [27] have enabled learners to engage in real-time dialogue with international peers, thus enhancing both language proficiency and cultural awareness.

Despite this progress, many studies treat language gains as proxies for intercultural growth, with limited focus on how learners develop empathy, adaptability, or critical cultural awareness. Moreover, such platforms may not be universally accessible due to regional internet restrictions, infrastructure limitations, or language barriers. In the Chinese context, global social media like Facebook and WhatsApp remain inaccessible without VPNs [12], restricting their utility for authentic intercultural practice.

By contrast, mobile apps like HelloTalk, which are accessible and culturally embedded, offer asynchronous and synchronous communication channels for intercultural exchange. Research has demonstrated HelloTalk’s efficacy in improving linguistic skills [28,29], but its impact on IC development remains underexplored.

### 2.3 Mobile-assisted intercultural competence in Chinese EFL education

China’s education policy encourages technology-enhanced language learning, as reflected in *The National Standards of Teaching Quality for Undergraduate English Majors*. However, most mobile learning applications used in China – such as WeChat, QQ, and vocabulary apps – primarily serve domestic audiences and emphasize language form rather than intercultural function [30–32]. Consequently, students, particularly in under-resourced regions, face limited opportunities to interact with speakers from different cultural backgrounds [33].

This digital divide is both technological and pedagogical. While first-tier universities may have access to international exchange programs or AI-driven simulations, many EFL learners in second- and third-tier cities still rely on low-cost, accessible tools like HelloTalk [34]. These mobile platforms reduce technical barriers while offering practical avenues for authentic intercultural exposure.

Unlike traditional platforms, HelloTalk connects users to global speakers via text, voice, and video, all without requiring VPNs or expensive equipment. It allows Chinese students to participate in intercultural communication anytime, anywhere, fostering learner autonomy and cultural adaptability in informal settings.

### 2.4 Summary and research gap

While prior studies have established foundational links between IC and MALL, key gaps persist. First, theoretical models such as Byram’s model often lack cultural adaptability to Chinese EFL contexts. Second, empirical evidence on HelloTalk’s role in IC development remains limited, with existing research focusing primarily on language gains. Furthermore, most MALL-IC studies emphasize Western or technologically advanced settings, neglecting underrepresented learner populations. This study addresses these gaps by examining HelloTalk’s potential to foster IC among Chinese EFL students, particularly those with constrained access to emerging technologies, thus offering contextually grounded theoretical and pedagogical insights.

## 3 Methodology

### 3.1 Participants

This study was conducted at a public university in a third-tier city in China. Participants were 105 third-year undergraduates (aged 21–23) enrolled in two classes of the elective course Intercultural Communication, which aims to develop students’ intercultural competence for academic, professional, and social purposes.

The experimental group included 50 students (22 males, 28 females), and the control group had 55 students (25 males, 30 females). They majored in fields such as finance, accounting, marketing, and computing. All participants had passed the College English Test Band 4 (CET-4), scoring between 430 and 550 (out of 710), equivalent to roughly 65–77 on a 100-point scale – indicating a generally comparable level of English proficiency suitable for intercultural communication tasks.

### 3.2 Research instruments

This study employed two instruments to assess students’ intercultural competence. First, the Assessment of Intercultural Competence of Chinese Students (AIC-CCS) [35] was used. This 28-item questionnaire, specifically designed for Chinese EFL learners, covers six dimensions including self-knowledge, knowledge of others, attitudes, intercultural communicative skills, intercultural cognitive skills, and awareness. It adopts a 5-point Likert scale and has demonstrated strong reliability (Cronbach’s alpha = 0.92 overall; 0.78–0.88 for subscales) and wide applicability in previous studies [36,37].

In addition, semi-structured interviews were conducted with volunteers from the experimental group to gain qualitative insights into their experiences using HelloTalk. Participants were asked how the App contributed to their intercultural competence and what they perceived as its strengths and weaknesses in facilitating intercultural communication. To ensure comfort and expression accuracy, the interviews were conducted in Mandarin and lasted approximately 20–25 minutes.

### 3.3 Research procedure

Before the study commenced, ethical approval was granted by the Ethics Committee at School of Foreign Languages for International Business, Hebei Finance University, on August 15, 2024. The study was carried out during the Fall 2024 semester (September to December), spanning 15 weeks, with a 90-minute session per week.

**Fig 1:**
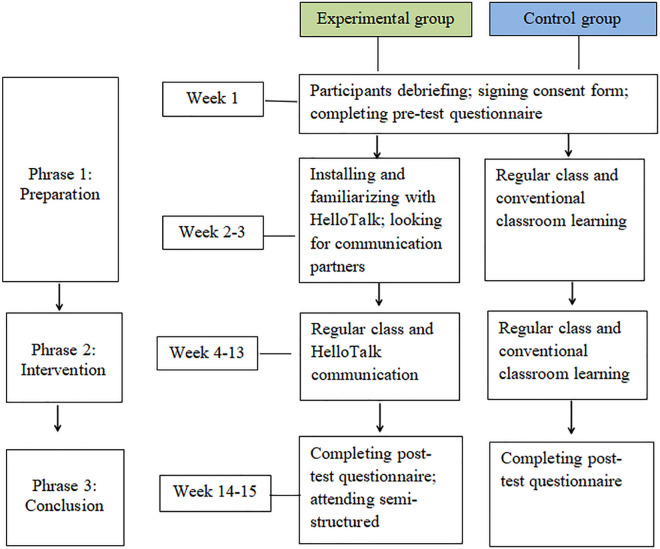
Research procedure.

The study was conducted in three phases over 15 weeks (see Fig 1).

Phase 1: Preparation (Weeks 1–3). Participants were informed of the study’s objectives and procedures, and all signed consent forms allowing the use of their data. In Week 1, both groups completed the pre-test (AIC-CCS) to assess baseline intercultural competence. During Weeks 2–3, the experimental group installed and explored the HelloTalk app, connecting with international users for intercultural communication.

Phase 2: Intervention (Weeks 4–13). The experimental group received weekly intercultural communication instruction (60 minutes) followed by guided HelloTalk-based intercultural exchanges (30 minutes), focusing on themes like festivals, education, and food. The control group engaged in traditional classroom activities such as lectures, case studies, and videos, without using HelloTalk.

Phase 3: Conclusion (Weeks 14–15). Both groups completed a post-test (AIC-CCS) to assess changes in intercultural competence. Additionally, 32 volunteers from the experimental group participated in semi-structured interviews to share their experiences using HelloTalk.

### 3.4 Data analysis

The current study employed a mixed research design, and it is particularly suited for capturing both the measurable improvements in intercultural competence and the in-depth experiences of EFL learners using HelloTalk. This approach allows for a comprehensive understanding of the effectiveness and perceptions of mobile-assisted intercultural learning in the Chinese educational context. Quantitative data from pre- and post-tests were analyzed using SPSS 26.0. An independent samples t-test confirmed no significant baseline differences between the groups. Paired samples t-tests assessed within-group changes, while further independent samples t-tests compared post-test scores across the six AIC-CCS domains between groups.

For the qualitative analysis, interview data from the experimental group were analyzed using [38]’s thematic analysis framework, aligned with the six AIC-CCS domains. Transcripts were reviewed and coded collaboratively by the researchers, resulting in two key themes that reflected participants’ experiences and perceptions of the HelloTalk intervention. Representative quotes were translated from Mandarin into English. The integration of quantitative and qualitative data followed a concurrent triangulation design. Quantitative findings provided measurable evidence of improvement in intercultural competence, while qualitative data added depth by contextualizing these changes through participants’ lived experiences. Themes from the interviews were mapped onto the AIC-CCS domains to enhance interpretive validity and to identify convergences or divergences across datasets. This integrative process allowed for a more comprehensive understanding of how HelloTalk influenced different dimensions of intercultural competence.

## 4 Results

### 4.1 Quantitative results

#### 4.1.1 Pre-test comparability.

An independent samples t-test was conducted to compare the experimental group (n = 50) and the control group (n = 55) on the pre-test intercultural competence levels. As shown in Table 1, no statistically significant difference was found (t = −0.05, *p *> 0.05), indicating comparable baseline levels of intercultural competence and ensuring the validity of subsequent analyses.

**Table 1:** Results for pre-test questionnaires of both groups.

					95% Confidence Interval of the Difference				
		N	M	SD	Lower	Upper	*t*	df	Sig. (2-tailed)
Experimental group	Pre-	50	3.02	.51	−.19	.18	−.05	103	.96
Control group	Pre-	55	3.02	.45					

#### 4.1.2 Post-test gains.

To examine the changes in intercultural competence resulting from the intervention, paired-samples t-tests were conducted for both the experimental and control groups. The results, presented in Table 2, indicate that both groups demonstrated significant improvements in intercultural competence from pre-test to post-test.

**Table 2:** Paired samples T test of both groups.

		N	M	SD	*t*	df	Sig. (2-tailed)
Experimental group	Pre-	50	3.02	.51	−6.64	49	<.001
	Post-	50	3.64	.44			
Control group	Pre-	55	3.02	.45	−2.94	54	<.005
	Post-	55	3.31	.56			

As shown in Table 2, both groups demonstrated significant improvements in intercultural competence. The experimental group’s mean score increased from 3.02 (SD = 0.51) to 3.64 (SD = 0.44), with a t-value of −6.64 (*p* < 0.001), indicating a strong effect from the HelloTalk-based intervention. The control group also improved, with scores rising from 3.02 (SD = 0.45) to 3.31 (SD = 0.56), t = −2.95, *p* < 0.005. These results suggest that both mobile-assisted and traditional instruction were effective, though the experimental group showed a greater gain.

To further assess the effectiveness of the two interventions, the mean gain scores for both groups were calculated, representing the difference between the post-test and pre-test scores. An independent samples t-test was conducted to compare the post-test intercultural competence scores between the experimental and control groups. The results are shown in Table 3.

**Table 3:** Results for post-test questionnaires of both groups.

		N	M	SD	Mean gain	*t*	df	Sig. (2-tailed)
Experimental group	Post-	50	3.64	.44	0.62	3.31	103	<.005
Control group	Post-	55	3.31	.56	0.29			

The experimental group had a mean gain score of 0.62 after the HelloTalk intervention. In contrast, the control group had a post-test mean gain score of 0.29.The independent samples t-test revealed a significant difference between the two groups after intervention (*t* = 3.31, *df *= 103, p < 0.005), suggesting that the HelloTalk-based intervention led to a significantly larger improvement in intercultural competence compared to the conventional classroom learning used in the control group. These findings indicate that while both interventions resulted in improvements in intercultural competence, the experimental group showed a significantly greater increase, highlighting the greater effectiveness of the HelloTalk-based intervention in enhancing Chinese EFL students’ intercultural competence.

#### 4.1.3 Domain-specific improvements.

To further explore the development of Chinese EFL students’ intercultural competence across the six domains of the AIC-CCS framework as a result of the HelloTalk intervention, independent samples t-tests were conducted to compare the experimental group with the control group in the following domains: Knowledge of self, Knowledge of others, Attitudes, Intercultural communicative skills, Intercultural cognitive skills, and Awareness. The results are presented in Table 4.

**Table 4:** Results for IC difference between groups across six domains.

Domain	Group		N	M	SD	Mean gain	*t*	df	Sig. (2-tailed)
Knowledge of self	Experimental	Pre-	50	3.19	.64	0.49	1.94	103	.06
		Post-	50	3.68	.65				
	Control	Pre-	55	3.33	.83	0.11			
			Post-	55	3.44	.60			
Knowledge of others	Experimental	Pre-	50	2.24	.64	1.04	4.67	103	<.001
		Post-	50	3.28	.71				
	Control	Pre-	55	2.36	.57	0.30			
			Post-	55	2.66	.66			
Attitudes	Experimental	Pre-	50	4.10	.81	0.05	0.43	103	.67
		Post-	50	4.15	.66				
	Control	Pre-	55	4.07	.71	0.02			
			Post-	55	4.09	.78			
Intercultural communicative skills	Experimental	Pre-	50	3.03	.67	0.60	2.12	103	<.05
		Post-	50	3.63	.07				
	Control	Pre-	55	2.85	.08	0.52			
			Post-	55	3.36	.10			
Intercultural cognitive skills	Experimental	Pre-	50	2.85	.76	0.70	2.66	103	<.01
		Post-	50	3.55	.58				
	Control	Pre-	55	2.94	.65	0.25			
			Post-	55	3.20	.77			
Awareness	Experimental	Pre-	50	3.20	.81	0.54	1.40	103	.17
		Post-	50	3.74	.70				
	Control	Pre-	55	3.26	.85	0.28			
			Post-	55	3.54	.78			

Significant differences emerged in three of the six domains: Knowledge of Others (t = 4.67, *p *< 0.001), Intercultural Communicative Skills (t = 2.12, *p* < 0.05), and Intercultural Cognitive Skills (t = 2.66, *p* < 0.01), with the experimental group making greater gains – especially in Knowledge of Others. No significant differences were found in Knowledge of Self (*p* = 0.06), Attitudes (*p* = 0.67), or Awareness (*p* = 0.17), indicating comparable effectiveness across both groups in these areas. Attitudes had the highest overall scores in both groups, suggesting a strong initial willingness to engage interculturally, possibly limiting the intervention’s impact. Overall, the results indicate that HelloTalk is particularly effective in developing learners’ cognitive and communicative dimensions of intercultural competence, which benefit most from real, dialogue intercultural interactions.

### 4.2 Qualitative results

To complement the quantitative results and gain deeper insight into students’ experiences, semi-structured interviews were conducted with 32 participants from the experimental group. Thematic analysis revealed two key themes regarding the perceived impact of the HelloTalk intervention on intercultural competence and its effectiveness in facilitating intercultural communication.

#### 4.2.1 Perceived impact.

Participants consistently reported reported increased understanding of foreign cultures, further supporting the quantitative findings. Many emphasized that HelloTalk experience enabled them to engage with individuals from diverse cultural backgrounds beyond typical classroom exposure. As one participant (female student, Grade 2) noted:

The project introduced me to new cultures. I learned about Ramadan and Nasi Lemak from a Malaysian, and discovered Morocco is in Africa, not Europe. Unlike traditional classes focused on the US or UK, this experience broadened my global understanding through real intercultural exchanges.

Her perceptions were shared by many of her peers, who similarly emphasized that the cultural knowledge gained through authentic intercultural communication was more impactful. Another participant (male student, Grade 2) offered a similar perspective, further supporting these views:

The best thing about HelloTalk was learning through real conversations. Talking with an American friend about individualism and collectivism helped me understand the concepts better than in class. After all, these concepts can be pretty boring when just learned in class.

Moreover, consistent with the quantitative findings, many participants reported improved intercultural communication skills as a result of the HelloTalk-based intervention. Specifically, they stated that they had become more confident and effective in communicating with foreigners. For most, this was their first experience of independently interacting with someone from another culture, providing them with a valuable opportunity to practice and refine their skills. As one participant (male student, Grade 3) reflected:

HelloTalk helped me get better at talking with foreigners. At first, I didn’t know how to start, but chatting more made me better at expressing myself and understanding others. I talked with a guy from Spain about food, and it felt natural. I wasn’t afraid to ask questions or share my thoughts. The more we talked, the easier it became.

Furthermore, participants reported increased respect for others during intercultural communication. Many became more aware of the need to remain open-minded and considerate in cross-cultural exchanges. For example, one participant (female student, Grade 3) noted how discussing sensitive topics like religion with a peer from the Middle East helped them ‘appreciate differing viewpoints’. Another (male student, Grade 2) shared that interacting with people from diverse backgrounds taught them to ‘listen more attentively and practice patience’, especially when facing language barriers. These accounts suggest that HelloTalk not only supported the development of communication skills but also fostered a deeper understanding of mutual respect in intercultural contexts.

#### 4.2.2 Effectiveness of using HelloTalk.

Participants’ interviews revealed several strengths of using HelloTalk in developing intercultural competence. The most frequently mentioned advantage was the platform’s accessibility for intercultural connection. Since popular global platforms like Twitter, Facebook, and WhatsApp are restricted in China, HelloTalk provides an easy, VPN-free way for Chinese EFL students to connect with foreigners. One participant (female student, Grade 3) said:

I never thought I could talk with foreigners since I don’t have Facebook or Twitter. But through this project, I found free apps like HelloTalk that don’t need a VPN. HelloTalk is a great platform for English learners to improve language and cultural skills.

Many participants also valued the flexibility of communication with HelloTalk, which allowed them to chat anytime and anywhere, not just during the 30-minute class sessions. The continuous conversations outside class made them feel close to their partners, even like ‘old friends’.

Additionally, numerous participants found HelloTalk’s asynchronous communication – via text and voice messages – helped reduce their anxiety. Unlike face-to-face chats, they could take time to express themselves without pressure over grammar or vocabulary mistakes. As one participant (female students, Grade 2) shared:

HelloTalk makes me feel less nervous. When I talk face-to-face with a foreign teacher, I get anxious and struggle to find the right words. I also get upset about making grammar mistakes. But on HelloTalk, I don’t worry about that because my partner still understands me, even if I make typing errors.

HelloTalk’s asynchronous chats also let users quickly search online when they don’t understand something. This helps them respond better and makes communication smoother and more effective.

Apart from strengths, participants mentioned two main weaknesses of using HelloTalk for intercultural communication. The primary limitation was intermittent communication difficulties. In the early stages of conversations, replies were often delayed or even missing. This made communication uncertain and interrupted the flow, so the planned 30-minute sessions often didn’t lead to deep or meaningful exchanges.

Secondly, safety concerns were also expressed, especially by female participants. Some users showed inappropriate behavior, likely due to the anonymity of online platforms like HelloTalk. One female student said a man from Pakistan asked for her school address and offered to send flowers, making her feel uneasy and cautious about online chats.

## 5 Discussion

This study investigated the efficacy of mobile-assisted language learning (MALL) via HelloTalk in enhancing intercultural competence (IC) among Chinese EFL students, compared with traditional classroom instruction. The mixed-methods findings were integrated using a parallel comparison approach, where qualitative themes helped explain and expand upon the statistical trends observed. This synthesis provided both breadth and depth in evaluating the effectiveness of the HelloTalk intervention. Quantitative and qualitative results consistently demonstrate that HelloTalk-based interventions yield significantly greater improvements in IC, corroborating previous findings on the potential of mobile applications to facilitate intercultural development and language acquisition [25–27]. Notably, among the six AIC-CCS domains, the largest gains were observed in Knowledge of Others, alongside substantial improvements in Intercultural Communicative Skills and Intercultural Cognitive Skills. This pattern aligns with previous literature emphasizing that intercultural exchanges primarily expand cultural knowledge through authentic cross-cultural interaction [39,40]. The pronounced increase in Knowledge of Others also reflects the initial cultural knowledge gap common among Chinese EFL learners, underscoring the urgent need for opportunities that foster direct intercultural engagement.

Conversely, domains related to Knowledge of Self, Attitudes, and Awareness showed no significant change. The stability of Attitudes aligns with prior studies reporting high baseline willingness among Chinese EFL students to engage interculturally, leaving limited scope for measurable growth [41,42]. This suggests that while students are generally open and positive toward intercultural communication, targeted interventions should prioritize expanding their cultural knowledge base and communication skills. Thus, mobile-assisted platforms like HelloTalk appear particularly suited to addressing these specific competency gaps, reinforcing their strategic value in EFL pedagogy.

The second research question explored students’ perceptions of HelloTalk’s role in developing IC. Qualitative data substantiate the quantitative trends, revealing that students valued the App’s facilitation of authentic, peer-to-peer intercultural dialogue. This supports social constructivist perspectives emphasizing interaction as central to effective language and cultural learning [43,44]. HelloTalk’s affordances for informal, spontaneous communication fostered relationship-building and cultural insight acquisition beyond the constraints of traditional classroom settings. Such findings resonate with prior studies highlighting the importance of interpersonal relationships in cultivating intercultural competence [2].

In particular, students reported notable gains in communicative skills, specifically in employing context-appropriate questioning strategies and demonstrating greater respect for cultural differences. These align with Byram’s concept of Skills of Discovery and Interaction, which emphasize the ability to acquire and apply new cultural knowledge dynamically during real-time exchanges [16]. The enhanced skill in asking culturally sensitive questions suggests educators should focus on cultivating learners’ pragmatic competence in intercultural questioning to deepen understanding and facilitate meaningful dialogue – an insight consistent with previous mobile-mediated intercultural exchange research [45].

Students identified several key advantages of HelloTalk. First, the platform’s accessibility within China, unlike many Western social media apps restricted by VPN requirements, allowed seamless intercultural interaction. This addresses a critical barrier often overlooked in Mobile-Assisted Language Learning (MALL) studies involving Chinese learners [36,37,39]. Second, HelloTalk’s temporal and spatial flexibility enabled participation unconstrained by time zones or fixed schedules, a feature widely recognized as beneficial in MALL research [46–48]. Third, the App’s asynchronous communication mode reduced interactional pressure, creating a “safe and comfortable space” conducive to reflective, thoughtful exchanges [49, p.622], corroborating findings in other mobile communication contexts [50]. This environment likely facilitates deeper cultural sensitivity by allowing learners time to process and respond mindfully.

Despite these strengths, two major challenges emerged. Difficulty in establishing sustained connections was prominent, consistent with prior observations of mismatched engagement levels and asynchronous communication pitfalls [51–53]. Addressing this requires instructor-led guidance on leveraging HelloTalk’s matching algorithms and encouraging detailed profile completion to foster compatible and motivated pairings, thus enhancing reciprocity and exchange quality [54]. The second challenge concerns the potential for deception inherent in open social platforms. Unlike instructor-mediated exchanges, the freedom to initiate contacts on HelloTalk exposed students to risks of misrepresentation. This underexplored issue necessitates pedagogical interventions that train students in critical profile evaluation, cautious information sharing, and detection of suspicious behaviors, supported by ongoing instructor monitoring to maintain a secure learning environment.

## 6 Conclusion

This study shows that HelloTalk effectively enhances intercultural competence among Chinese EFL students, particularly in cultural knowledge and communication skills. Its flexible, asynchronous design reduces interaction pressure, creating a more comfortable learning environment. HelloTalk’s accessibility without VPN restrictions is a key advantage in China, enabling spontaneous intercultural exchanges.

However, challenges like connectivity problems and risks of misrepresentation require careful pedagogical guidance and ongoing supervision. Educators should integrate such tools thoughtfully to maximize benefits and minimize risks. Future research should investigate how psychological and cultural factors—such as motivation, emotions, and identity—affect the impact of mobile-assisted intercultural learning. Exploring these deeper influences will advance understanding of how mobile technology supports intercultural competence development.

## Supporting information

S1 File Questionnaire. (PDF)

S2 File Minimal Data Set. (XLS)
