# Supplementary material for: Mobile-Assisted intercultural competence development: The role of HelloTalk in Chinese EFL education
Source: PLoS One. 2025 Jul 17;20(7):e0328660. doi: 10.1371/journal.pone.0328660 (PMC12270120; doi:10.1371/journal.pone.0328660)
Supplement: S1 File — (PDF) [file pone.0328660.s001.pdf]

Assessment of Intercultural Competence of Chinese College Students
（AIC-CCS）

This questionnaire is designed to collect your self-evaluation of your intercultural competence. According to your understanding, rate the following items from A to E about your objective and universal intercultural competence. We need your true thoughts. Thank you!

Please read the following items and rate yourself using the scale below:
(A) very low (B) low (C) average (D) high (E) very high

1. Knowledge of self
(1) understanding native history
(2) understanding native social norms
(3) understanding the native sense of values
2. Knowledge of others
(1) understanding foreign knowledge of history
(2) understanding foreign social norms
(3) understanding the foreign sense of values
(4) understanding foreign cultural taboos
(5) understanding foreigners' speech
(6) understanding basic concepts of intercultural communication
(7) understanding successful intercultural communication strategies
3. Attitudes
(1) willingness to learn from those who differ from one's self and culture
(2) willingness to respect foreigners' lifestyles and customs
(3) willingness to learn foreign languages and cultures well
4. Intercultural communicative skills
(1) the skill of consulting with foreigners when misunderstandings occur
(2) the skill of communicating with foreigners using body language or other nonverbal communication when it is difficult to communicate using language
(3) the skill of successfully communicating with foreigners
(4) the skill of treating foreigners politely
(5) the skill of avoiding offending foreigners with inappropriate words and behavior
(6) the skill of avoiding prejudice against foreigners
(7) the skill of avoiding violating foreigners' privacy

(8) the skill of having intercultural sensitivity
(9) the skill of understanding different perspectives when encountering different cultural affairs

5. Intercultural cognitive skills
(1) the skill of acquiring knowledge of other cultures from foreigners
(2) the skill of learning intercultural communication strategies
(3) the skill of learning how to manage cultural conflicts

6. Awareness
(1) realizing cultural differences and similarities when communicating with foreigners
(2) realizing the differences in cultural identity when communicating with foreigners
(3) judging cultural situations from both one’s own and the other’s cultural perspective
